# Production and characterization of tearless and non-pungent onion

**DOI:** 10.1038/srep23779

**Published:** 2016-04-06

**Authors:** Masahiro Kato, Noriya Masamura, Jinji Shono, Daisaku Okamoto, Tomoko Abe, Shinsuke Imai

**Affiliations:** 1 Basic Technology Development Division, Central Research & Development Institute, House Foods Group Inc., Yotsukaido, Chiba 284-0033, Japan; 2 Plant Breeding Institute Co., Ltd., Kuriyama-cho, Hokkaido 069-1511, Japan; 3 Nishina Center for Accelerator-Based Science, RIKEN, Wako, Saitama 351-0198, Japan

## Abstract

The onion lachrymatory factor (LF) is produced from *trans*-*S*-1-propenyl-L-cysteine sulfoxide (PRENCSO) through successive reactions catalyzed by alliinase (EC 4.4.1.4) and lachrymatory factor synthase (LFS), and is responsible for the tear inducing-property and the pungency of fresh onions. We developed tearless, non-pungent onions non-transgenically by irradiating seeds with neon-ion at 20 Gy. The bulbs obtained from the irradiated seeds and their offspring bulbs produced by selfing were screened by organoleptic assessment of tear-inducing property or HPLC analysis of LF production. After repeated screening and seed production by selfing, two tearless, non-pungent bulbs were identified in the third generation (M3) bulbs. Twenty M4 bulbs obtained from each of them showed no tear-inducing property or pungency when evaluated by 20 sensory panelists. The LF production levels in these bulbs were approximately 7.5-fold lower than those of the normal onion. The low LF production levels were due to reduction in alliinase activity, which was a result of low alliinase mRNA expression (less than 1% of that in the normal onion) and consequent low amounts of the alliinase protein. These tearless, non-pungent onions should be welcomed by all who tear while chopping onions and those who work in facilities where fresh onions are processed.

Bulb onion (*Allium cepa* L.) is one of the most widely cultivated vegetables in the world. Its annual production in 2013 was estimated to be approximately 85.8 million tons, which ranked third after tomato and watermelon in quantity in a list of cultivated vegetable crops worldwide^1^.

The most conspicuous feature of the onion is its tear-inducing, pungent properties, which is not welcome by many consumers. Numerous tips to avoid tearing when chopping onions have been proposed, such as lighting a match, wearing sealed goggles with a foam seal, chilling the onion, or chopping it under water, etc^2^. Furthermore, the market for low-pungency onions, such as those achieved by low sulfur uptake or growth in sulfur deficient soils, is expanding in Europe and the United States. The consumption of low-pungency onions now accounts for 15–25% of total onion consumption in the United States^3^. Those low-pungency onions, however, have poor storage characteristics because of their relatively high moisture content.

Pungency has been correlated with production of the lachrymatory factor (LF)^4^, which was identified as propanethial *S*-oxide almost 40 years ago by Brodnitz and Pascale^5^. LF is formed from 1-propenyl sulphenic acid by lachrymatory factor synthase (LFS)^6^. The 1-propenyl sulphenic acid is a putative reaction product derived from 1-propenyl cysteine sulfoxides ((*E*)-PRENCSO) by alliinase (EC 4.4.1.4, syn. cysteine sulfoxide lyase). In other words, the LF is produced through the successive enzymatic reactions that occur upon onion tissue disruption.

This suggests that the formation of the LF is under control of specific gene products, and that development of tearless, non-pungent onion may be possible via genetic modification (GM), mutagenesis, or conventional plant breeding procedure. Unlike existing low-pungency onions, onions in which the function of enzymes involved in the LF forming reactions are suppressed should not exert tear-inducing property and pungency, irrespective of their moisture content. In fact, onions in which alliinase or lachrymatory factor synthase were suppressed through RNA interference have been reported^7^,^8^. In most countries, however, newly developed transgenic plants have to go through a difficult approval process before they may be produced commercially.

Recently, heavy-ion beams have been used widely to generate mutants of higher plants, because, in contrast to other mutagenic techniques, they induce mutations with high frequency at a relatively low dose, and various phenotypes may be obtained without other plant characteristics being affected^9^,^10^,^11^. To date, unique and various flowers and crops such as Verbena^12^, Torenia^13^, rice^14^,^15^ have been produced, but this has not yet occurred in vegetables. Mutants produced by ion-beam radiation are not considered transgenic, and are expected to gain better acceptance by the consumers.

In this study, we used a neon-ion beam to produce tearless and non-pungent onion using the Japanese onion cultivar ‘Super-Kitamomiji’ with long-day (day-length sensitivity: approximately 14 hours), long-storage (storage length: 6–7 months), medium to large sized (bulb weight: 180–260 g), and high-pungency (pyruvic acid content: 5–10 μmol/g fw) characteristics. We report the production and characterization of this new tearless, non-pungent onion.

## Results

### Screening of tearless, non-pungent onion bulbs

**Figure 1:**
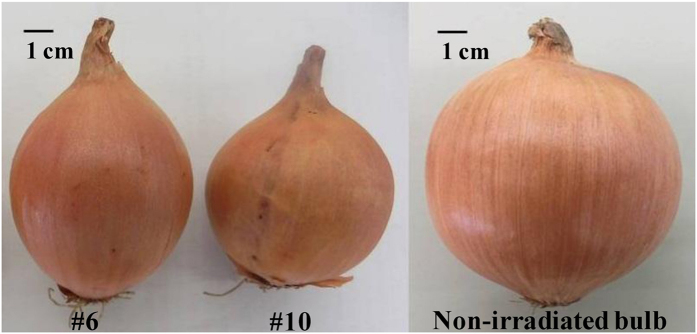
A photograph of the tearless, non-pungent onion bulbs (#6, #10) and non-irradiated onion bulb (‘Sapporo-Ki’). The bar shown in the photograph indicates 1 cm.

We irradiated about 1,500 dry seeds of the popular Japanese onion cultivar s of the popular Japanese onions (Fig. 1). A total of 1,450 M1 seeds were planted and grown in the usual manner to obtain 457 mature bulbs, from which 9 bulbs exhibiting weaker or little lachrymatory factor production as determined by organoleptic assessment and HPLC analysis were selected and used for M2 seed production by selfing. About 350 M2 seeds were harvested and planted, from which 197 bulbs were obtained and analyzed for alliinase protein by Enzyme-Linked Immunosorbent Assay (ELISA). Eighteen bulbs that showed less than 1/10 of an ELISA signal shown by a normal (non-irradiated) onion were selected and planted for M3 seed production by selfing. Of the 3,250 M3 seeds harvested, 1,078 seeds were planted to obtain 466 bulbs, from which two bulbs (#6 and #10) were finally selected based on the amount of PRENCSO (>3.26 μmol/g FW) remaining after tissue disruption and organoleptic examinations of the pungency. They were the offspring of a single irradiated seed. Of the total of 666 M4 seeds obtained from #6 and #10 bulbs by selfing, 200 seeds each were sown. They grew normally and yielded 158 (#6) and 68 (#10) bulbs, which were morphologically indistinguishable from normal (non-irradiated) onion bulbs (‘Sapporo-Ki’) (Fig. 1). These M4 bulbs were used for the following analyses.

### Organoleptic evaluation of tear-inducing property and pungency

To examine the degree of tear-inducing property and pungency of the M4 onion bulbs, 20 each of the M4 bulbs were crushed and tested by the 20 sensory panelists. All panelists responded that none of the tested bulb samples showed tear-inducing property or pungency (data not shown). Because of the absence of pungency, many panelists reported perception of sweetness normally masked by the pungency. Furthermore, the distinctive flavor of onion did not linger at all in the oral cavity after tasting. These results shows that the M4 bulbs from #6 and #10 lines have characteristics very different from those of the normal onion bulbs.

### Chemical analysis of tear-inducing property and pungency

**Figure 2:**
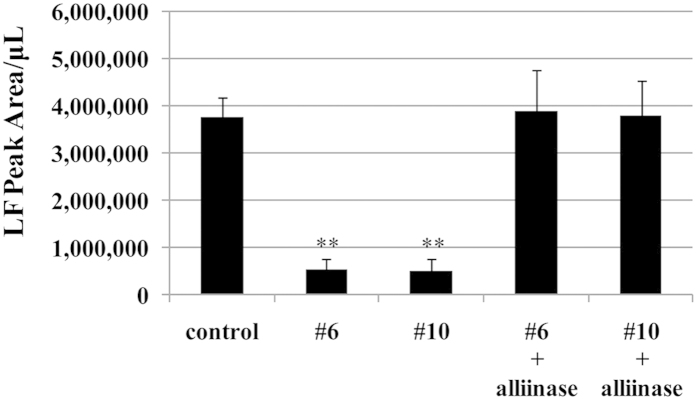
Analysis of LF production in onion bulbs (control, #6 and #10). The LF productions are presented as LF peak area per μL of supernatant. The control represents the normal onion (‘Sapporo-Ki’) grown in the same field and by the same cultivation method as #6 and #10. Data are mean ± SD, n = 5 (control), n = 19 (both #6 and #10), n = 18 (both #6 + alliinase and #10 + allinase). Significant differences between control and the test samples by Dunnett’s test (P < 0.01) are indicated by **.

**Figure 3:**
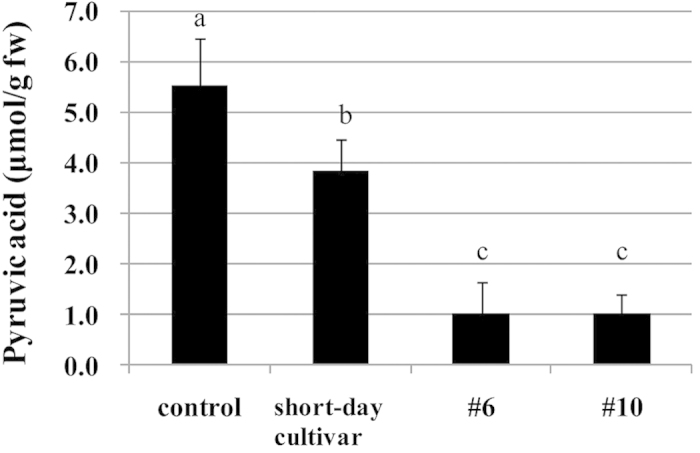
Analysis of pyruvic acid in onion bulbs (control, short-day onion, #6 and #10). Pyruvic acid concentrations are presented as μmol per g fresh onion weight. The control represents the normal onion (‘Sapporo-Ki’) grown in the same field and by the same cultivation method as #6 and #10. The short-day cultivar represents short day onions collected from 5 different areas (Chiba, Nagasaki, Saga, Kagoshima and Shizuoka) in Japan. Data are mean ± SD, n = 5 (control), n = a total of 5 (one from each areas (short), n = 19 (both #6 and #10). Significant differences by Tukey–Kramer test (P < 0.01) are indicated by different letters.

The amounts of the lachrymatory factor (LF) and pyruvic acid were measured after bulb tissue disruption. LF is responsible for both the tear-inducing property and the pungency of onion^4^. Pyruvic acid has long been used as a simple and quick measure of pungency of onion, because it is a breakdown product of PRENCSO by alliinase, and under the presence of lachrymatory factor synthase (LFS), its amount positively correlates with the amount of LF produced. The LF levels in two lines (#6 and #10) were significantly reduced by approximately 7.5-fold compared with those of the normal onion (Fig. 2). The pyruvic acid levels were significantly decreased by 5.5-fold compared with those of the normal onion, and were decreased by 3.8-fold compared with those of short-day onion cultivars (Fig. 3). These results are consistent with the results of organoleptic evaluation. The production levels of LF (<1,000,000 Peak Area/μL) and pyruvic acid (<2 μmol/g fw) in the two lines (#6 and #10) were the lowest of all non-transgenic onion cultivars we have ever analyzed (>2,000,000 Peak Area/μL and >2.2 μmol/g fw, respectively). The pyruvic acid levels in the two lines (#6 and #10) were significantly decreased by 2.4-fold compared with the literature values reported for the low-pungency onions such as ‘Vidalia’ grown under low sulfur fertilizer or on sulfur deficient soils^16^,^17^,^18^. From these results, we concluded that the two lines (#6 and #10) were the least pungent onions among the cultivars authors are aware of.

An addition of purified alliinase protein (12.5 Units) resulted in an increase in LF production in #6 and #10 to the levels of the control onion (Fig. 2). This result suggested that the activities of LFS in #6 and #10 were at the same level in the control onion. Therefore, the decreased levels of pyruvic acid production in #6 and #10 upon tissue disruption may be explained by either a reduced level of PRENCSO or alliinase activity, or both.

### Quantification of PRENCSO levels

**Figure 4:**
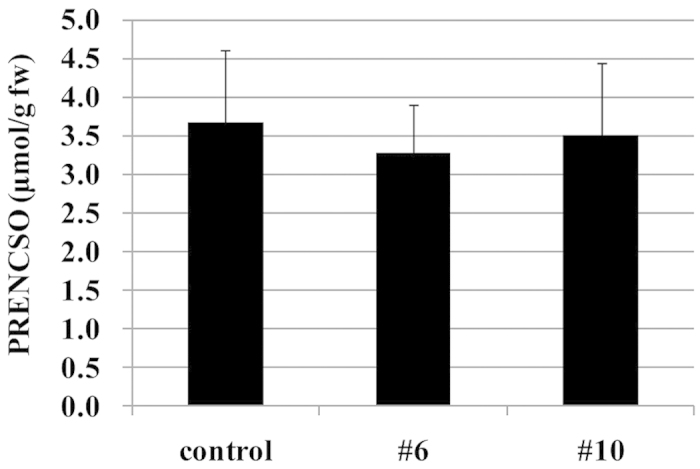
Analysis of PRENCSO in heated onion bulbs (control, #6 and #10). PRENCSO concentrations were presented as μmol per g fresh onion weight. The control was in the normal onion (‘Sapporo-Ki’) grown in the same field and by the same cultivation method as #6 and #10. Data are mean ± SD, n = 5 (control), n = 19 (both #6 and #10). No significant differences were observed between control and the test samples (#6, #10) by Dunnett’s test.

PRENCSO levels in heated tissues of #6, #10 and the normal onion as measured by HPLC-PDA were between 2.5 and 4.6 μmol/g (Fig. 4), which were within the normal physiological range reported for onions^19^,^20^.

### Evaluation of alliinase activity

**Figure 5:**
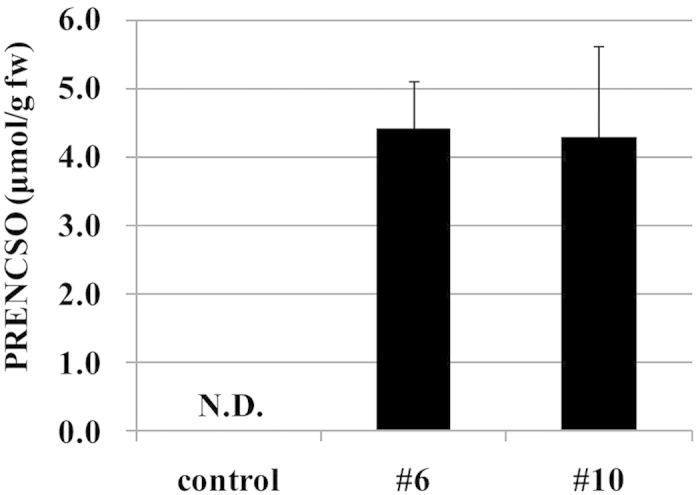
Analysis of PRENCSO in unheated onion bulbs (control, #6 and #10). PRENCSO concentrations are presented as μmol per g fresh onion weight. The control represents the normal onion (‘Sapporo-Ki’) grown in the same field, the same cultivation method as #6 and #10. Data are mean ± SD, n = 2 (control), n = 8 (#6) and n = 9 (#10). N.D., not detected.

The PRENCSO content in the unheated tissues of #6 and #10 bulbs were between 3.0 and 5.4 μmol/g, compared with 0 μmol/g in the control (Fig. 5). These results strongly suggested that PRENCSO in the tissues of #6 and #10 bulbs was not cleaved by the alliinase, and that the alliinase activity may be significantly reduced.

### Quantification of alliinase protein expression levels

**Figure 6:**
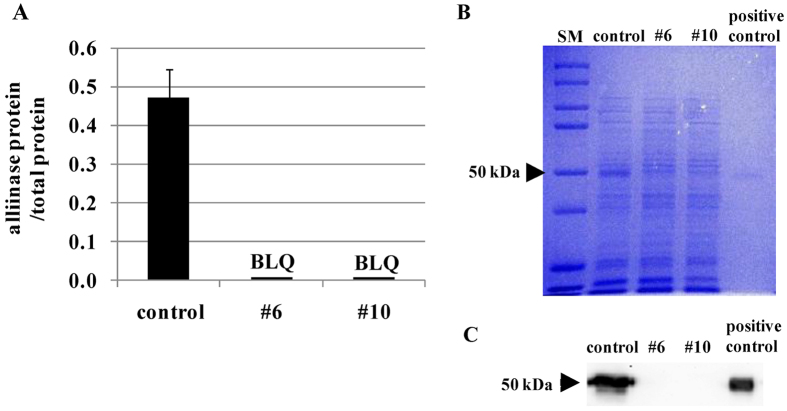
Analyses of protein expression of alliinase in onion bulbs (control, #6 and #10). Protein expression of alliinase in control, #6 and #10 are compared by ELISA (**A**), SDS-PAGE analysis (**B**) and western blot assay (**C**). The control represents the normal onions (‘Sapporo-Ki’) grown in the same field and by the same cultivation method as #6 and #10. The alliinase purified from fresh garlic cloves by using a hydroxyapatite column and a concanavalin A–sepharose 4B column was used as the positive control. Precision Plus Protein Kaleidoscope Prestained Protein Standards was used as the SM (size marker). The results of ELISA are presented as mean ± SD, n = 5 (control), n = 19 (both #6 and #10). BLQ, below the limit of quantitation.

The alliinase protein levels in #6 and #10 bulbs were below the limit of quantitation of the ELISA (Fig. 6A). The SDS-PAGE and the Western blot of #6 and #10 bulbs showed no alliinase protein (Fig. 6B,C). Thus, the reduction in alliinase activity was proven to be due to the reduction in the alliinase protein levels.

### Quantification of alliinase mRNA expression levels

**Figure 7:**
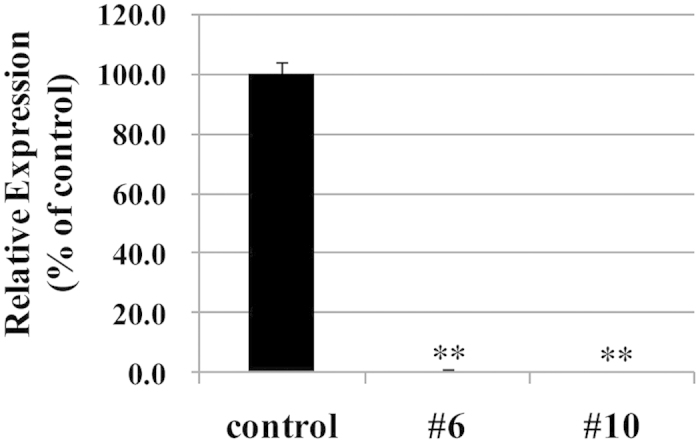
Analysis of alliinase mRNA levels in onion bulbs (control, #6 and #10). The mRNA expression levels of alliinase were quantified by real-time PCR. The expression levels are normalized to the amount of *ubiquitin* transcript and presented in percentage relative to the mean expression level in the control bulbs. Data are mean ± SD, n = 3 (each sample). Significant differences between control and the test samples by Dunnett’s test (P < 0.01,) are indicated by **.

Quantitative real-time RT-PCR (qRT-PCR) showed that the expression levels of alliinase mRNA in #6 and #10 bulbs were less than 1% of that in the normal onion respectively (Fig. 7). These results showed that reduced alliinase protein levels in #6 and #10 bulbs were due to low alliinase mRNA expression levels during the transcription process.

### Soluble Solids measurement

**Figure 8:**
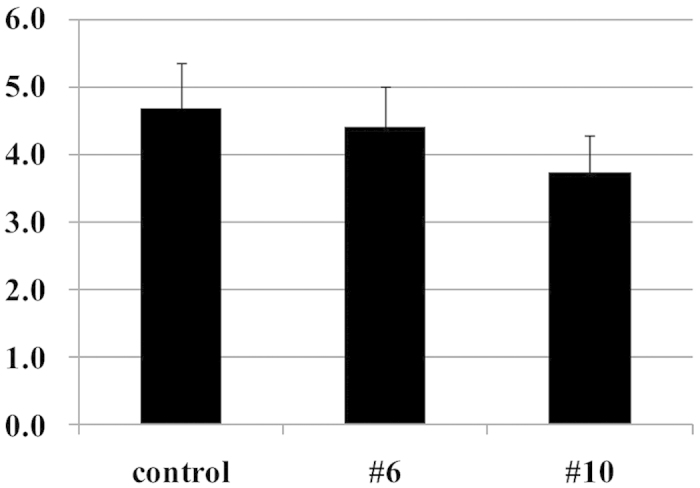
Soluble Solids measured in onion bulbs (control, #6 and #10). The control represents the normal onion (‘Sapporo-Ki’) grown in the same field and by the same cultivation method as #6 and #10. Data are mean ± SD, n = 5 (control), n = 19 (both #6 and #10). No significant differences were observed between control and the test samples (#6, #10) by Dunnett’s test.

The soluble solids levels in #6, #10 bulbs and the normal onion bulbs as measured by the refractometer were between 2.8 and 5.7, and no statistical differences were observed among the three groups (Fig. 8). These results are consistent with the sweetness perceived in #6 and #10 bulbs during organoleptic evaluations.

## Discussions

In this study, we succeeded in producing two lines of tearless, non-pungent onion by using heavy-ion beam mutagenesis. Tearless and non-pungent characteristics in these two lines were due to the significant reduction of alliinase mRNA expression levels. PRENCSO in these lines was not broken down by alliinase and remained after tissue disruption because of the absence of alliinase activity. However, it still remains unresolved how and why the alliinase mRNA expression levels in these two lines are so dramatically lower.

One possibility is that the mutation occurred in an alliinase gene that is expressed in the bulb. To date, two types of alliinase genes, bulb type^21^,^22^,^23^ and root type^24^,^25^, have been isolated from *Allium cepa*. Expression of the root type genes in onion bulbs is not detectable or, at most, very low^24^,^25^. Therefore, it is more likely that the mutation occurred in the bulb type gene, if it was the gene that was affected by the neon-ion beam irradiation. Bulb alliinase genes have been mapped to a few loci in the onion genome. King *et al*.^26^ revealed two loci of the alliinase markers located in a distance of 6.9 cM^26^. Van Heusden *et al*.^27^ mapped an alliinase gene in the AFLP linkage group assigned to the chromosome 4 of *A. cepa*^27^. Further in the intraspecific cDNA map reported by Martin *et al*.^28^, two alliinase gene loci were located at a distance in a linkage group unassigned to chromosomes^28^. On the basis of the analysis of the two genetic maps produced by King *et al*.^26^ and Martin *et al*.^28^, along with the results of Tyr-FISH mapping of an alliinase clone, Khrustalevaa *et al*.^29^ assumed the unassigned linkage group possessing the alliinase loci belonged to chromosome 4^29^. Information on the size of deletions caused by neon-ion beam irradiations at a dose of 20 Gy with LET at 63 keV/μm is not available, but at 15 Gy with the same LET, the irradiations reportedly caused deletions of sizes ranging from 2 bps to 12 bps in various genes, and induced frame-shift mutations in rice^30^,^31^,^32^. In another study based on amplicon sequencing approach, it was suggested that deletions from less than 200 kbps to larger than 2 Mbps had occurred in wheat irradiated at 50 Gy with the same LET^33^. Although such deletions may occur in multiple sites on the chromosomes at the same time, chances that simultaneous deletions occurred at the two loci of the bulb alliinase genes located 6.9 cM apart should be small. As suggested by King *et al*.^26^, the bulb alliinase gene mapped to one of the two loci could be a pseudogene. Thus, the likely explanation would be that the neon ion beam irradiation caused a deletion in the only one transcriptionally active bulb alliinase gene located at one of the loci on chromosome 4 and rendered the gene inactive. Another possibility is that the mutation occurred in the transcription factor that controls expression of alliinase genes, but little information on the transcription factor of alliinase is currently available.

In the next study, we will perform differential analysis of transcript expression using high-throughput RNA sequencing (RNA-seq) between tearless, non-pungent onions and normal onions to look into the above hypotheses more in detail. Further, we will develop an F2 population by crossing normal onions with the two lines obtained in this study and determine the inheritance of low alliinase activity in the two lines to reveal how many genes are responsible for the low alliinase activity.

We believe that these tearless, non-pungent onions not only will wipe tears away from the kitchen and the food processing facilities, but also will add a new dimension to the enjoyment of onion recipes around the world.

## Methods

### Materials

All chemicals used were purchased from Wako Pure Chemical (Osaka, Japan) or Kanto Chemical (Tokyo, Japan) unless otherwise noted. All solvents used were chromatography grade. Onion seed (cultivar ‘Super-Kitamomiji’) was purchased from Plant Breeding Institute Co., Ltd. (Hokkaido, Japan). Short-day onions of unknown cultivar grown in Chiba, Nagasaki, Saga, Kagoshima and Shizuoka in Japan were purchased from a local store. The normal yellow onion (cultivar ‘Sapporo-Ki’) grown in the same field and by the same cultivation method as the tearless, non-pungent onion was used as the control. Alliinase was prepared from fresh garlic cloves by using a hydroxyapatite column and a concanavalin A-Sepharose 4B column as previously described by Imai *et al*.^34^. The purified alliinase was dissolved in 500 mM sodium phosphate buffer (pH 7.0) containing 10% glycerol and 25 μM pyridoxal phosphate at 250 units/mL.

### Production and screening of tearless, non-pungent onion

Approximately 1500 dry seeds of onion cultivar Super-Kitamomiji were irradiated with neon-ions at a dose of 20 Gy under atmospheric pressure (RIKEN RI-beam factory (RIBF), Saitama, Japan). The LET of ions corresponded to 63 keV/μm.

A total of 1,450 M1 seeds were planted and grown to bulb maturity in the standard cultivation method adopted in Hokkaido, Japan, including the cultivation schedule, planting distances, and the use of fertilizers. After removal of the brown coats, about 5–10 g of the outer scales was excised longitudinally from the M1 bulbs and subjected to the first screening based on lachrymatory factor production as determined by organoleptic assessment and HPLC analysis as described below. Remaining portion of the selected bulbs was grown in a greenhouse for seed production by selfing. Second and third screenings were carried out using M2 and M3 onion bulbs based on the Enzyme-Linked Immunosorbent Assay (ELISA) and the analysis of PRENCSO remaining after tissue disruption as described below. For these stages of screening, a horizontal slice of about 1 mm thickness was excised from the mature bulb at about 1/2 to 2/3 of the bulb height, and about 400 to 600 mg of the third to fourth scale from the center was used for the analyses. The bottom half of the selected bulbs including the basal plate was grown in the greenhouse for seed production by selfing. The seeds obtained from selected M3 bulbs and the seeds of ‘Sapporo-Ki’ were planted and grown together in the same field plot to obtain M4 and control bulbs, which were subjected to the analyses described below.

### Organoleptic evaluation of tear-inducing property and pungency

Twenty panelists evaluated tear-inducing sensation and pungency arising from masticating fresh bulb tissues in the mouth. The panelists were asked to rinse the oral cavity with water between each evaluation.

### Quantification of lachrymatory Factor (LF)

The lachrymatory factor (LF) was measured using an HPLC. Four hundred mg of fresh onion bulb tissue was crushed with or without 12.5 units purified alliinase using a micro pestle for 20 seconds, and immediately after centrifugation at 15,000 rpm at 4 °C for 1 min, 1 μL of the supernatant was applied to the HPLC (LC-10AT, Shimadzu) equipped with a Senshu Pack ODS column (250 mm × 4.6 mm, 5 μm, Senshu Scientific) and a UV detector (254 nm). The mobile phase was 30% methanol containing 0.05% TFA (LC-MS grade). The flow rate was set to 0.6 mL/min and the column oven temperature was set at 35 °C.

### Quantification of pyruvic acid

The pyruvic acid was measured using a microplate reader (DS Pharma Biomedical) based on the method described by Anthon and Barrett^35^. Briefly, 600 mg fresh onion bulb tissue was crushed with 600 μL distilled water using a Mixer Mill MM 300 (QIAGEN) for 6 min at 30 Hz, and held for 10 min at room temperature. After centrifugation at 15000 rpm at 4 °C for 10 min, 20 μL of the supernatant was applied to a 96 well plate. Approximately 30 min after crushing, 43 μL of distilled water and 66 μL of 0.25 g/L DNPH in 1M HCl were added to the supernatant in the 96 well plate and the plate was rolled gently for 10 min at 37 °C. After 10 min, 66 μL of 1.5 M NaOH was added. The absorbance at 515 nm was measured by the microplate reader. Standards were prepared from a 40 mM sodium pyruvate solution, ranging in concentration from 0.04 to 6 mM.

### Quantification of PRENCSO

PRENCSO levels in heated and unheated (raw) onion bulb tissues were determined as described by Fritsch and Keusgen^36^ with a slight modification. To 600 mg of fresh onion bulb tissue, 600 μL distilled water was added, and either heated for 1 hour at 60 °C, or held for 1 hour at room temperature. The tissues were crushed using the Mixer Mill MM 300 (QIAGEN) for 6 min at 30 Hz, and after centrifugation at 15000 rpm at 4 °C for 10 min, 1 μL of the supernatant was applied to the HPLC (Alliance 2695, Waters) equipped with a Senshu Pack ODS column (250 mm × 4.6 mm, 5 μm, Senshu Scientific) and a photodiode array detector (210–800 nm). The mobile phase was 0.05% trifluoroacetic acid in water. The flow rate was set to 0.6 mL/min and the column oven temperature was set at 35 °C.

### Enzyme-Linked Immunosorbent Assay

A sandwich enzyme-linked immunosorbent assay (ELISA) was used for determination of alliinase protein as described by Kamata *et al*.^37^. An antibody against alliinase was raised in rats by Operon Biotechnologies (Tokyo, Japan) using a purified onion alliinase protein. Affinity-purified anti-alliinase antibody was used as the capture layer in a sandwich ELISA. For the detection antibody, the anti-alliinase antibody was biotinylated using Biotin Labeling Kit-NH2 according to the manufacturer’s protocol (Dojindo Mol). A microplate reader (DS Pharma Biomedical) was used for the detection. The quantification range of the ELISA was 0.3–4.8 μg/mL of purified garlic alliinase protein as the standard. Alliinase protein expression levels are presented as the ratio relative to the total protein quantified using a Protein Assay Kit (Bio-Rad) with bovine gammaglobulin (SIGM) as the standard.

### Western Blot analysis

Total protein was extracted from 500 mg of onion bulb tissue, which had been frozen at −80 °C, with 500 mL of PBS buffer and quantified using a protein assay (Bio-Rad) with bovine gammaglobulin (SIGM) as the standard. Alliinase, as the positive control, was purified from fresh garlic cloves. The purified alliinase was also quantified using a protein assay (Bio-Rad) with bovine gammaglobulin (SIGM) as the standard. Nine μg of the extracted total protein and 175 ng of the purified alliinase in Laemmli Sample Buffer (Bio-Rad) were loaded onto a 10% polyacrylamide gel and electrophoresed. The protein was stained by Coomassie Brilliant Blue (CBB) or electrically blotted onto a polyvinylidene difluoride membrane (Bio-Rad) using a Trans-Blot SD Cell (Bio-Rad) following the manufacturer’s instructions. The blot was blocked by 5% skim milk solution and incubated overnight with an alliinase polyclonal antibody raised in a rat. Excess antibody was removed by two 30 min washes in Tween-PBS buffer, and the membrane was incubated for 1 h with an anti-rat IgG solution. Again, excess antibody was removed by three 30 min washes in Tween-PBS buffer, and the alliinase protein was detected using the HRP Conjugate Substrate Kit (Bio-Rad) according to the manufacturer’s instructions. The membranes were imaged on a ChemiDoc XRS (Bio-Rad).

### Quantification of mRNA expression levels

Total RNA was prepared from onion bulb tissues, which had been frozen with liquid nitrogen, by using RNeasy Plant Mini Kit (QIAGEN), and reverse-transcribed using ReverTra Ace (TOYOBO). To determine mRNA expression levels, real-time quantitative RT-PCR analysis was performed on a 7900 HT Fast Real-Time PCR Systems (Applied Biosystems) using SYBR green fluorescence signals.

The oligonucleotide primer sets of ubiquitin and alliinase were designed from the sequence data in the GenBank database as follows: Ubiquitin (GenBank accession number AA451588; Fwd: 5′-ACGATTACACTAGAGGTGGAGAGCTC-3′; Rev: 5′-CCTGCAAATATCAGCCTCTGCT-3′), alliinase (GenBank accession number L48614, AF121962, FJ446579, Z12621, AF124405; Fwd: 5′-AATGAGACCTCCATCCCCATC-3′; Rev: 5′-TCGAAACCCTCTCCACTTTG-3′). The mRNA expression levels are presented as the ratio relative to a putative *ubiquitin* used in each experiment as the internal standard gene. The amplification conditions consisted of 1 min at 95 °C, 45 cycles of 15 sec at 95 °C, 15 sec at 55 °C and 30 sec at 72 °C and 5 min at 72 °C.

### Soluble Solids Measurement

Soluble Solids was measured using a Digital Refractometer RX-5000α (ATAGO). Briefly, 600 mg fresh onion bulb tissues were crushed with 600 μL distilled water using the Mixer Mill MM 300 (QIAGEN) for 6 min at 30 Hz. After centrifugation at 15000 rpm, for 10 min at 4 °C, 100 μL of supernatant was applied.

### Statistical analyses

Data are presented as mean ± SD. The data were assessed for statistical significance by Dunnett’s test or Tukey–Kramer’s multiple comparison tests. Differences were considered significant when p < 0.01.

## Additional Information

**How to cite this article**: Kato, M. *et al*. Production and characterization of tearless and non-pungent onion. *Sci. Rep.* **6**, 23779; doi: 10.1038/srep23779 (2016).
